# A Sequence-Specific Nicking Endonuclease from *Streptomyces*: Purification, Physical and Catalytic Properties

**DOI:** 10.1155/2013/287158

**Published:** 2013-08-21

**Authors:** Peechapack Somyoonsap, Vichein Kitpreechavanich, Somchai Pornbanlualap

**Affiliations:** ^1^Interdisciplinary Program in Genetic Engineering, Kasetsart University, Bangkok 10900, Thailand; ^2^Department of Microbiology, Kasetsart University, Bangkok 10900, Thailand; ^3^Department of Biochemistry, Kasetsart University, Bangkok 10900, Thailand

## Abstract

A sequence-specific nicking endonuclease from *Streptomyces* designated as DC13 was purified to near homogeneity. Starting with 30 grams of wet cells, the enzyme was purified by ammonium sulfate fractionation, DEAE cellulose, and phenyl-Sepharose chromatography. The purified protein had a specific activity 1000 units/mg and migrated on SDS-PAGE gel with an estimated molecular weight of 71 kDa. Determination of subunit composition by gel filtration chromatography indicated that the native enzyme is a monomer. When incubated with different DNA substrates including pBluescript II KS, pUC118, pET-15b, and pET-26b, the enzyme converted these supercoiled plasmids to a mixture of open circular and linear DNA products, with the open circular DNA as the major cleavage product. Analysis of the kinetic of DNA cleavage showed that the enzyme appeared to cleave super-coiled plasmid in two distinct steps: a rapid cleavage of super-coiled plasmid to an open circular DNA followed a much slower step to linear DNA. The DNA cleavage reaction of the enzyme required Mg^2+^ as a cofactor. Based on the monomeric nature of the enzyme, the kinetics of DNA cleavage exhibited by the enzyme, and cofactor requirement, it is suggested here that the purified enzyme is a sequence-specific nicking endonuclease that is similar to type IIS restriction endonuclease.

## 1. Introduction

Restriction enzymes recognizing unique DNA sequences are indispensable tools in molecular biology. Among three different types of restriction enzymes that have been described, type II endonucleases are the most useful enzymes for gene cloning because they recognize 4–8 bp sequences and cleave the DNA within or in close proximity to the recognition site [1]. More than 3500 different type II restriction endonucleases with 240 unique specificities have been currently identified [2]. Restriction endonucleases working with methyltransferases form the basis of RE (restriction modification) systems of prokaryotes to protect them against the invasion of foreign DNA [3]. 

Type II endonucleases consist of structurally and mechanistically diverse subclasses of enzymes, each with a different mechanism of sequence recognition, DNA cleavage, and organization of quaternary structure [4]. The orthodox type IIP enzymes are the most studied and include the majority of enzymes such as *Eco*RI, *Eco*RV, and *Bam*HI commonly used in molecular biology [5]. These enzymes typically exist in solution as a homodimer, bind to a symmetrical palindromic 4–8 bp sequence as a dimer, and cleave phosphodiester bonds on both strands of DNA within the recognition site [2]. The crystal structure of 16 of these enzymes including *Eco*RI, *Bam*HI, *Bgl*II, and others had been determined [6–8]. Despite the lack of sequence similarity at the primary sequence level, these enzymes display an *α*/*β* architecture, where the central sheet formed by a five-strand mixed *β*-sheet is flanked on both sides by *α*-helices. Because a single DNA-binding cleft is formed at the interface between two monomers, the homodimeric enzymes bind only to one recognition site. Other subclasses of enzymes, including type IIE, IIF, and IIS, differ from type IIP in that they must simultaneously bind to two (or more) copies of their recognition site to cleave DNA [9]. Although they interact with two recognition sites, type IIE and IIS enzymes cleave only one recognition site, whereas type IIF enzymes cleave both.

Type IIS enzymes are composed of two-domain proteins which consist of a DNA-binding domain and a catalytic domain [10]. *Fok*I is the best characterized type IIS enzyme. *Fok*I molecules exist in solution as monomer and dimerization of two *Fok*I monomers cannot occur in the absence of DNA. Once the first monomer is bound to its asymmetrical recognition sequence, its catalytic domain becomes exposed and free to dimerize with the catalytic domain of a second monomer that is bound to another recognition sequence [11]. Dimerization of two *Fok*I molecules is mediated through interaction between the catalytic domains of two monomers. Because *Fok*I molecules contain one active site per monomer, dimerization of two catalytic domains is essential for cleavage of phosphodiester bonds on both strands of DNA at the recognition site. The dimerized catalytic domains which contain two catalytic sites subsequently rotate to cleave both strands of DNA at fixed distances away from the targeted recognition site. 

Type IIE enzymes are also two-domain proteins and must simultaneously bind two palindromic recognition sites, one being the actual site of cleavage while the other acts as the allosteric effector [12]. Unlike type IIS, the native type IIE enzymes form homodimer in solution. The best characterized type IIE enzymes are *Eco*RII and *Nae*I [13, 14]. Each of these enzymes has been shown to consist of a DNA effector-binding domain and a catalytic domain. As with type IIE, because there is only one catalytic site per monomer, dimerization of two catalytic domains is required for cleavage of phosphodiester bonds on both strands of DNA. In the absence of DNA, the enzyme forms an “inactive” dimer because the effector-binding domain is blocking the catalytic site. Upon binding of an allosteric-binding domain of a monomer in the dimeric enzyme to a recognition site (the effector site), the catalytic domain of a second monomer becomes activated, binds to the other recognition sites (the substrate site), and cleaves both strands of DNA.

 Type IIF endonucleases are one-domain proteins and, with the exception of *Sgr*AI, exist in solution as tetramers. The best characterized type IIF enzymes are *Cfr*10I, *Ngo*MIV and *Bse*643I [15–17]. The two dimers are arranged back-to-back so that each binds to a separate recognition site on the opposite face of the tetramer. These enzymes cleave both recognition sites within or near both recognition sites. Cooperativity is mediated at the interface between two dimers so that the enzyme cleaves both recognition sites concertedly without release of a one-site cleavage product.

Although more than 3500 different type II restriction endonucleases with 240 unique specificities have been currently identified, only two naturally existing nicking endonucleases, N.*Bst*SEI and N.*Bst*NBI, that recognize the DNA sequence, 5′-GAGTC-3′, are currently available [18]. Because of their potential applications in isothermal DNA amplification, nicking endonucleases have recently captivated interest from researchers. In an isothermal DNA amplification, the combined repetitive cleavage of a phosphodiester bond in the recognition sequence in one strand of the double-strand DNA by the nicking endonuclease followed by strand-displacement synthesis by DNA polymerase results in linear amplification of one strand of the DNA molecule [19]. Because restriction endonucleases are valuable tools in gene cloning and the mechanisms of DNA recognition/cleavage are fundamental to biochemistry, this study describes purification and characterization of the physical and catalytic properties of a sequence-specific nicking endonuclease from *Streptomyces *DC13. 

## 2. Materials and Methods

### 2.1. Bacterial Strain, Growth Condition, and Reagents

Forty strains of unidentified *Streptomyces* were isolated from soil samples collected from deciduous dipterocarp forest in Thailand. Cells were maintained on nutrient agar (15 g agar, 5 g peptone, 5 g NaCl, 2 g yeast extract, and 1 g beef extract per liter) and grown at 37°C. For enzyme purification, a single white colony was inoculated into modified LB (10 g peptone, 5 g yeast extract, 5 g NaCl, and 1 g glucose (pH 7.2) per liter) and grown aerobically by shaking at 250 rpm at 37°C for 72 hours into late logarithmic phase before harvesting by centrifugation. Plasmids used for DNA cleavage assays, including pBluescript II KS (StrataGen), pUC118, pET-15b, and pET-26b (Novogen), were propagated in *E. coli* DH5*α* and purified by the standard alkaline lysis method [14]. Restriction enzymes were purchased from New England Biolabs, USA.

### 2.2. Determination of the 16S rDNA of *Streptomyces*

*Streptomyce*s strain DC13, which contains the nicking endonuclease activity, was identified by sequencing of the 16S rRNA gene. The 16S rRNA gene, approximately 1500 base pairs (bp), was amplified from the *Streptomyces* DNA by PCR, using forward (5′-TCACGGAGAGTTTGATCCTG-3′) and reverse (5′-AAGG AGATCCAGCCG CA-3′) primers [20]. *Streptomyces* genomic DNA was purified by the protocol as described by Kieser et al. [21]. Amplification reaction was performed in a 20 *μ*L reaction mixture containing 1x PCR buffer, 0.125 pM of forward and reverse primers, 0.2 mM dNTP, 1.5 mM MgCl_2_, 0.03 U of *Taq* DNA polymerase, and 10% DMSO. PCR was performed using the following conditions: initial denaturing at 94°C for 4 min; 30 cycles of denaturation at 94°C for 1 min, annealing at 55°C for 1 min, and additional extension at 72°C for 1.5 min and additional extension at 72°C for 10 min. PCR product was electrophoresed on a 1% agarose gel and stained with ethidium bromide. The 1.5 kb PCR fragment corresponding to the 16S rDNA gene was purified using QIAquick PCR purification kit (Qiagen, CA, USA) according to the manufacturer's instructions and sequenced by the terminator cycle sequencing method. The obtained partial nucleotide sequence of 16S rRNA gene was analyzed and compared with 16S rRNA genes of other microorganisms deposited in the GenBank by the BlastN program [22].

### 2.3. Purification of the Endonuclease

Thirty grams (wet weight) of *Streptomyces *DC13 cells were suspended in 50 mM buffer A (150 mM Tris-HCl (pH 8.0), 150 mM NaCl, 0.5 mM EDTA, 1 mM PMSF, 0.5% Tween 20, and 0.5% NP40), broken by sonication for 20 min, and centrifuged at 10,000 ×g for 30 min. To remove nucleic acids from the supernatant, streptomycin sulfate was added at a final concentration of 1.5%. The mixture was stirred for 20 min and centrifuged at 10,000 g for 30 min. Solid ammonium sulfate was added to the supernatant to give 60% saturation at 4°C. The mixture was stirred for 30 min and centrifuged at 10,000 ×g for 30 min. The protein pellet was collected, suspended in buffer A, and dialyzed four times against 4 liters of 25 mM potassium phosphate (pH 7.5). The dialyzed protein was applied to a DEAE-cellulose DE32 column (2.5 cm × 8.5 cm) that had been preequilibrated with buffer B (50 mM potassium phosphate (pH 7.5), 1 mM EDTA, and 10 mM *β*-mercaptoethanol). Proteins were eluted with a gradient of 0 to 200 mM NaCl in buffer B. Active fractions eluted at low salt were pooled and concentrated by ultrafiltration, using an Amicon pressure cell. The concentrated protein was purified further by a one-step fast protein liquid chromatography (FPLC) system, using Phenyl-Sepharose 6 Fast Flow (0.5 cm × 5.0 cm) column (Pharmacia). The column was preequilibrated with 50 mM potassium phosphate (pH 7.0) containing 1 M (NH_4_)_2_SO_4_. Proteins were eluted with a gradient of 1 to 0 M (NH_4_)_2_SO_4_ in 50 mM potassium phosphate buffer at a flow rate of 2 mL/min. 

### 2.4. Apparent Molecular Mass Determination

The molecular mass of the purified enzyme was determined by gel filtration FPLC, using a HiPrep 16/60 Sephacryl S-200HR column (Pharmacia). A solution (0.5 mL) containing 0.5 mg/mL of purified endonuclease, 10 units of pyruvate kinase (237 kDa), 3 mg/mL conalbumin (75 kDa), 4 mg/mL ovalbumin (43 kDa), 50 mM KH_2_PO_4_ (pH 7.0), and 150 mM NaCl was applied to a column which had been equilibrated with the same buffer but lacking the enzymes. The flow rate was 1 mL/min and the elution profiles were monitored at 280 nm by UV absorption or enzyme assay. 

### 2.5. DNA Cleavage Assay

Enzyme activity was measured by incubating the endonuclease with supercoiled plasmid or lambda DNA in buffer R (10 mM Tris-HCl (pH 8.0), 10 mM MgCl_2_, 50 mM NaCl, and 5 mM *β*-mercaptoethanol). After 30 min of incubation, the reactions were quenched by addition of 1/3 volume of loading dye solution containing 0.5% SDS and heating at 80°C for 5 min. Cleavage products were analyzed on 1 or 1.5% agarose gels. One unit of enzyme activity was defined as the amount of enzyme needed to convert 1 *μ*g of supercoiled DNA into an open circular or linear DNA in one hour at 37°C. The unit of endonuclease activity was quantified by titrating against known units of commercial *Eco*RI (New England Biolabs). Protein concentration was determined by the Bradford method using BioRad protein assay reagent (BioRad, USA) and BSA as the standard. 

### 2.6. DNA Binding Assay

 The 500 bp DNA fragments containing DNA sequence upstream from the CoE1 origin of replication were amplified by PCR, using pBluescript as template. The forward primer used was T_7_ universal primer (5′-GTAATACGACTCACTATAGGC-3′) and the reverse primer was T_500_ (5′-GGATAACCGTATTACCGCCTTTG-3′). PCR reaction was performed in a 100 *μ*L reaction mixture containing 20 ng of pBluescript II KS and 0.08 mM of each primer. The mixture was incubated at 93°C for 5 min then subjected to 35 cycles of 1 min denaturation at 93°C, 40 sec of annealing at 60°C, and 1 min 40 sec of extension at 72°C. PCR product was precipitated by sodium acetate/ethanol and redissolved in TE buffer. DNA binding assay was carried out by incubating 500 bp DNA fragment with the purified endonuclease at 37°C for 10 min in 10 mM Tris-HCl (pH 8.0), 10 mM MgCl_2_, 50 mM NaCl, and 5 mM *β*-mercaptoethanol. The samples were analyzed on a 1.2% agarose gel. 

### 2.7. Kinetics of Supercoiled Plasmid Cleavage

The kinetics of DNA cleavage was analyzed by incubating 2–12 *μ*g of endonuclease with 1–6 *μ*g of plasmid DNA in buffer R (total volume = 120 *μ*L). Plasmids purified by the alkaline lysis method in our experiments consisted of 78–85% supercoiled DNA. At various time points, aliquots were removed and quenched by addition of 1/3 volume of loading dye solution containing 0.5% SDS. Cleavage products were analyzed on 1 or 1.5% agarose gel electrophoresis. 

## 3. Results

### 3.1. Identification of Isolated *Streptomyces* by Sequencing of 16S Ribosomal RNA Gene

 The 1.5 kb 16S rRNA gene was amplified by PCR and sequenced at the 5′-end. Analysis of the 600 bp nucleotide sequence obtained indicated that it contained a highly variable *α*-region. Comparison of this sequence to the 16S rRNA gene in GenBank using the BlastN program showed that it shared the highest similarity (98.7%) to *S. hygroscopicus*, *S. sparsogenes,* and *S. acidiscabie*. At present, no known sequence-specific nicking endonuclease has been isolated from *Streptomyces*. 

### 3.2. Purification of the Endonuclease

**Figure 1:**
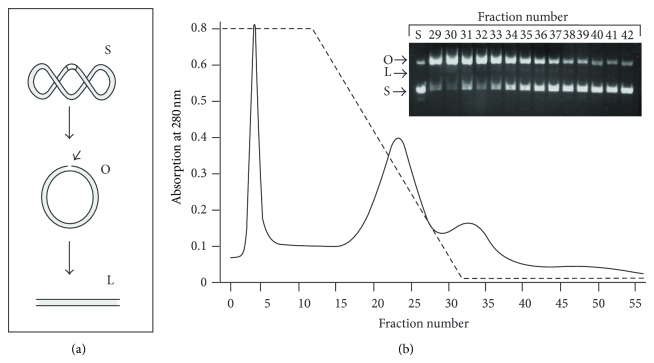
Schematic diagram of the plasmid cleavage reaction and chromatographic profile of proteins eluted from a Phenyl-Sepharose column. (a) Diagram showing various DNA products resulting from cleavage of supercoiled plasmid by the endonuclease. Various forms of pBluescript are indicated: S, supercoiled DNA; O, open-circular DNA; L, linear DNA. (b) Chromatographic profile of the protein eluted with a linear gradient of 1 to 0 M (NH_4_)_2_SO_4_ in 50 mM potassium phosphate buffer at a flow rate of 2 mL/min. The dashed line (- - - -) represents the gradient of ammonium sulfate. The inset shows the DNA cleavage activity of proteins eluted in fractions 29 to 42 determined as described in Section 2.

**Figure 2:**
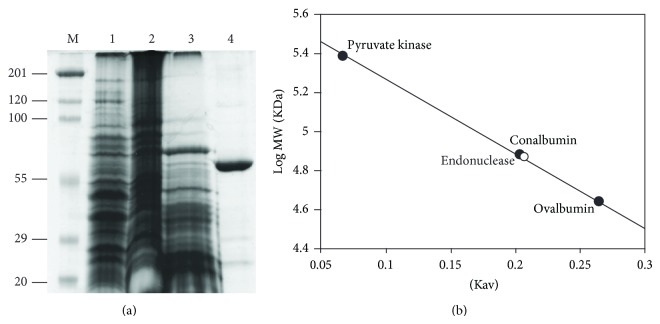
Analysis of the purified endonuclease by polyacrylamide gel electrophoresis and gel filtration chromatography. (a) M: standard protein markers; lane 1: cell-free extract; lane 2: 0–60% ammonium sulfate fraction; lane 3: protein purified by DEAE-cellulose chromatography; lane 4: protein purified by hydrophobic interaction chromatography. (b) Molecular weight determination by gel filtration chromatography on a Sephacryl S-200HR column.

A sequence-specific nicking endonuclease was purified from thirty grams of *Streptomyces *cells. The purification protocol involved ammonium sulfate fractionation, DEAE-cellulose chromatography, and hydrophobic interaction chromatography. Although the endonuclease did not bind or bound weakly to a DEAE-cellulose column, other contaminant proteins including nonspecific nucleases bound to the column and were removed in the process, resulting in approximately 5-fold increases in a specific activity. When applied to a hydrophobic interaction column, proteins resolved into three major peaks. The third peak (fractions 26–40) was found to contain most of the endonuclease activity and cleaved supercoiled pBluescript II KS into mostly an open circular DNA with a minor linearized DNA product also formed in the reaction (Figure 1, *inset*). Proteins in these fractions were pooled and concentrated by ultrafiltration. SDS-PAGE analysis of the purified protein showed that it consists of a single band (Figure 2(a)) that migrated with an estimated molecular weight of 71 kDa from the plot of log MW versus mobility (data not shown). The purification protocol resulted in an apparently pure protein with a specific activity of 1,000 units/mg (Table 1). When loaded onto a Sepharcyl-200 gel filtration column, the enzyme eluted with an apparent molecular weight of ~70 kDa (Figure 2(b)), indicating that the native enzyme is a monomer. 

### 3.3. Substrate Specificity of the Endonuclease Purified from *Streptomyces* DC13

At the beginning of these experiments, both lambda DNA and supercoiled plasmids were used as DNA substrates to detect the endonuclease activity of *Streptomyces*. However, because plasmid DNA, but not lambda DNA, was cleaved by the enzyme, supercoiled pBluescript II KS was subsequently used as the substrate to assay the endonuclease activity. After the protein had been purified to near homogeneity, the recognition sequence/cleavage site of the endonuclease on pBluescript was located using other supercoiled plasmids such as pUC118, pET-15b, and pET26b as substrates. Although these plasmids share sequence similarity to pBluescript such as a colE1 origin of replication, they carry other genes such as kanamycin-resistance and *lac *repressor genes which are not found in pBluescript. 

**Figure 3:**
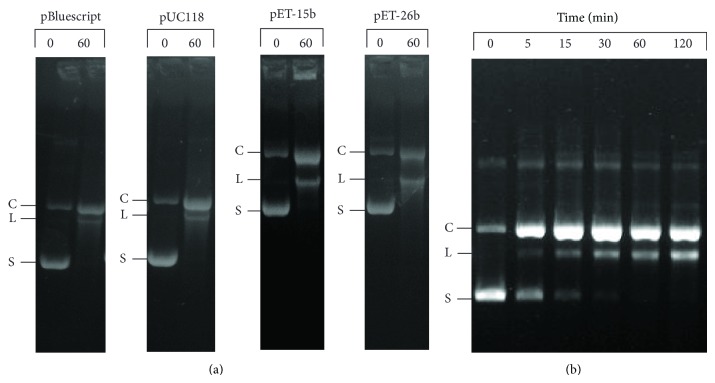
Substrate specificity and kinetics of plasmid cleavage by the endonuclease. (a) Plasmids DNAs including pBluescript II KS, pUC118, pET-15b, and pET-26b as indicated were incubated with the purified endonuclease in buffer R for 0 and 60 min as described in Section 2. (b) Endonuclease (12 *μ*g) was incubated with supercoiled pBluescript (6 *μ*g) in buffer R at 37°C (total volume 100 *μ*L). At various time points as indicated, aliquots of reaction mixture were withdrawn and analyzed on 1% agarose gel. Supercoiled DNA (S); an open circular DNA (O); linearized DNA (L).

**Figure 4:**
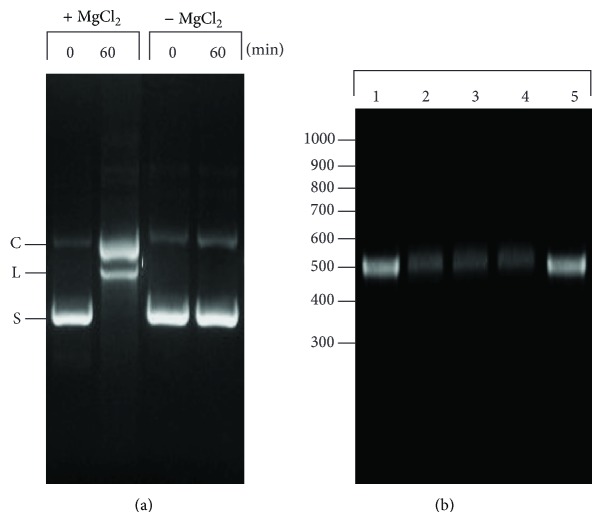
Cofactor requirement and gel shift assay. (a) The purified protein (2 *μ*g) was incubated with supercoiled pBluescript II KS (1 *μ*g) in buffer R with (+) and without (−) 10 mM MgCl_2_ for 60 min at 37°C before being analyzed on an agarose gel. (b) The purified 500 bp fragment (1 *μ*g) was incubated with increasing amount of protein in buffer R (total volume 50 *μ*L) for 15 min at 37°C and was analyzed on 1.2% agarose gel. Lanes 1 and 5 are size standard of a purified 500 bp DNA fragment. The 500 bp fragment (1 *μ*g) was incubated with the purified enzyme at 5 *μ*g (lane 2), 7 *μ*g (lane 3), and 8.8 *μ*g (lane 4), respectively.

Plasmids purified by the alkaline lysis in our experiments consisted of 78–85% supercoiled DNA when quantified on a densitometer. When supercoiled pBluescript II KS was incubated with the enzyme, the majority of the cleavage product formed was open circular rather than linearized DNA. Even after 60 minutes of incubation, only a fraction of linearized DNA product was observed (Figure 3(a)). These data suggested that only one strand of double-stranded DNA at the recognition site was hydrolyzed by the enzyme, generating an open circular DNA containing nick(s). When pUC118, pET-15b, and pET-26b were used as DNA substrate, a similar cleavage pattern was observed: most of the cleavage product observed was open circular rather than linearized DNA. To investigate the kinetics of DNA cleavage, the time course of product formation was followed. When incubated with the enzyme, supercoiled pBluescript II KS was rapidly converted into an open circular DNA, which was followed by a slower conversion to linear DNA. The open circular DNA accumulated in the reaction and did not completely convert to linear DNA even after 120 min of incubation (Figure 3(b)). This result showed that the cleavage activity of endonuclease is unlike most type IIP restriction endonucleases such as *Eco*RI and *Bam*HI. These enzymes completely cleave supercoiled plasmids containing one recognition site into the linearized DNA with transient formation of an open circular DNA as an intermediate [23, 24]. When supercoiled pBluescript II KS was incubated with the enzyme for 12 hours, the amount of linearized DNA (2.6 kb) observed was only 15–20% (data not shown). Furthermore, the linearized DNA formed in the reaction did not further degrade to smaller DNA fragments with prolonged incubation. These data suggested that the nicking activity of the enzyme is sequence specific. If the nicking endonuclease was nonspecific and nicked the DNA randomly, then prolonged incubation would result in further degradation of linearized DNA into smaller fragments. Similar to type II restriction endonucleases, the DNA cleavage of the nicking endonuclease required magnesium as a cofactor. In the presence of magnesium chloride, the enzyme cleaved supercoiled plasmid into open circular and linear DNA (Figure 4(a)). When magnesium chloride was omitted in the reaction mixture, however, no DNA cleavage activity was observed. When a 500 bp PCR fragment amplified from upstream region of the ColE1 origin of replication was incubated with the enzyme in a gel shift assay, this fragment was retarded by the enzyme (Figure 4(b)). Other DNA fragments containing the ColE1 origin of replication were also retarded by the enzyme (data not shown).

## 4. Discussion

### 4.1. Properties and DNA Substrate Specificity of the Endonuclease

 A sequence-specific nicking endonuclease was isolated from *Streptomyces* DC13 and purified to near homogeneity. Comparison of the nucleotide sequence of the 16S rRNA gene of *Streptomyces* DC13 to other known *Streptomyces* deposited in the GenBank showed that it shares the highest sequence similarity (98.7%) to *S. hygroscopicus*, *S. sparsogenes,* and *S. acidiscabie*, respectively. The purified enzyme has a molecular mass of 71 kDa and exists in solution as a monomer. When various supercoiled plasmids, including pBluescript, pUC118, pET-15b, and pET-26b, were incubated with the enzyme, the majority of cleavage product formed was open circular rather than linearized DNA. This observation suggests that the enzyme appears to introduce one or a few specific nicks onto the plasmid, converting the superhelical DNA into open circular DNA. When a second nick is introduced onto the opposite strand of DNA at the same recognition site by the endonuclease, open circular DNA was converted to linear DNA. The DNA cleavage activity of the enzyme requires magnesium as a cofactor. Because the purified endonuclease appears to cleave pBluescript, pUC118, pET-15b, and pET-26b at least once, a computer-aided search of homologous nucleotide sequences among these plasmids revealed they shared sequence homology near or at the CoE1 origin of replication. When a 500 bp PCR fragment amplified from upstream region of the ColE1 origin of replication was incubated with the enzyme in a gel shift assay, this fragment was retarded by the enzyme. This result indicates that the endonuclease binds specifically to the 500 bp fragment. The exact location and sequence on the 500 bp fragment where endonuclease binds remain to be determined. A similar gel retardation experiment had been used to demonstrate that type IIS *Mly*I and *Ple*I endonucleases bind specifically to the DNA fragment containing GAGTC, its recognition sequence [25]. 

### 4.2. DNA Cleavage Pattern of the Nicking Endonuclease Is Similar to *Ple*I Endonuclease

Analysis of the kinetics of DNA cleavage showed that supercoiled pBluescript II KS was converted to linear DNA in two distinct steps: a rapid cleavage of one strand of double stranded DNA to form open circular DNA followed by a much slower cleavage of the opposite strand of double stranded DNA at the same site to form linear DNA (Figure 1(a)). After formation of the open circular DNA, two possible reaction pathways may follow. In one pathway, the enzyme either slowly translocated to the opposite strand of DNA or dimerized with a second monomer to form a dimer and then cleaved the opposite strand of double stranded DNA, generating a linearized DNA. Because the amount of open circular DNA peaked at 5 min and did not completely convert to linear DNA even after 120 min of incubation (Figure 3(b)), this step must occur at a very slow rate. In the second reaction pathway, the enzyme translocated along DNA to another recognition site somewhere else on the open circular DNA and cleaved one strand of the double stranded DNA, generating an open circular DNA with two nicks. Because agarose gel cannot separate an open circular DNA with one nick from that with two or more nicks, whether this cleavage reaction also occurred is unknown. 

The molecular weight, subunit composition, and kinetics of DNA cleavage of the purified endonuclease are markedly different from that of the orthodox type IIP such as *Eco*RI and *Bam*HI. Most type IIP enzymes are small proteins (~20–35 kDa), consist of one domain, and form dimer in solution in the absence of DNA. With *Eco*RI, supercoiled plasmid with one recognition site appears to be converted directly to linear DNA, although small amount of open circular DNA is also transiently formed as an intermediate in the reaction [23]. The DNA cleavage pattern of the purified endonuclease in this study is more similar to that observed with type IIS enzymes. The kinetics of DNA cleavage of supercoiled plasmid containing one recognition site by type IIS enzymes, including *Ple*I, *Myl*I, and a nicking endonuclease N.*Bst*NBI, had been investigated [25]. These enzymes recognize the same GAGTC sequence, but differ in the mechanism of DNA cleavage. Although both *Myl*I and *Ple*I cleave supercoiled plasmid sequentially, they form different DNA cleavage products. Cleavage of supercoiled plasmid by *Mly*I resulted in a complete conversion to linear DNA, although an open circular DNA was transiently formed as an intermediate. Cleavage of supercoiled plasmid by *Ple*I, however, resulted in a mixture of open circular and linear DNA products. Furthermore, the open circular DNA formed in the reaction accumulated and did not completely convert to linear DNA even after prolonged incubation. This DNA cleavage pattern is most similar to that observed with the purified endonuclease. With the nicking endonuclease N.*Bst*NBI, cleavage of supercoiled plasmid resulted in open circular DNA. No linear DNA cleavage product was observed. In addition, similar to the purified endonuclease, the type IIS enzymes also exist in solution as monomers.

Based on the kinetic of DNA cleavage, subunit composition, and the two-domain nature of proteins, various mechanisms of DNA cleavage had been proposed by Higgins et al. for *Fok*I, *Ple*I, and *Mly*I [25]. Structural studies of *Fok*I, a prototype of type IIS endonuclease, have shown that it is a two-domain protein consisting of a catalytic domain and a DNA-binding domain. Because only one catalytic site existed per monomer, hydrolysis of two phosphodiester bonds of double stranded DNA requires dimerization of two *Fok*I molecules. Prior to binding to the recognition site, however, dimerization cannot occur because the catalytic domain of the monomer is sequestered and cannot interact with each other [26]. When the DNA-binding domain of the first monomer binds to a recognition site, its catalytic domain becomes exposed and free to dimerize with catalytic domain of a second monomer that is bound to another recognition sequence. When dimerized, the catalytic site of each monomer becomes active and concertedly hydrolyzes the two phosphodiesters on both strands of DNA, resulting in mostly linear DNA product. Because cleavage of supercoiled plasmid by *Ple*I and *Mly*I resulted in a mixture of open circular and linear DNA products at the early stage of reaction, the monomers of these enzymes have been proposed to be catalytically active prior to dimerization. When the first monomer binds to a recognition site, it hydrolyzes the phosphodiester bond of one strand of DNA to generate open circular DNA. When this monomer dimerizes with a second monomer, hydrolysis of the phosphodiester of the second strand of DNA by the second monomer produces a linear DNA. The nicking enzyme N.*Bst*NBI produces mainly open circular DNA from supercoiled plasmid. This enzyme has been proposed to be a mutant of type IIS that cannot dimerize and is catalytically active as monomer [26].

Because the purified nicking endonuclease is a monomeric protein and cleaves supercoiled plasmid to form a mixture of open circular and linear DNA products, a possible DNA cleavage mechanism of this enzyme would be similar to that of *Ple*I endonuclease. Although it is unclear whether the purified endonuclease in this study is also a two-domain protein, it is interesting to note that the molecular weight of the purified enzyme is 71 kDa, approximately the same as most type IIS enzymes, *Fok*I (65.5 kDa), *Myl*I (65 kDa), and N.*Bst*NI (70.8 kDa) [25]. Trypsin digestion of *Fok*I monomer resulted in a 41 kDa amino terminal fragment that constitutes the DNA-binding domain and a 25 kDa carboxyl terminal fragment that constitutes the catalytic domain [26]. Most type IIP enzymes, however, are one-domain protein and thus have a smaller molecular weight (~20–35 kDa) [8, 27, 28] than the type IIS enzymes [25]. On the other hand, the mechanism of DNA recognition and cleavage of this enzyme may be entirely different from that of the type IIS enzymes. Further experimental data are needed to provide an insight into the catalytic mechanism of the purified enzyme. In addition to type IIS and nicking enzymes, other endonucleases are also known to introduce a specific nick into double stranded DNA. Most of these enzymes are involved in DNA metabolism such as DNA replication, repair, and recombination [29–32]. However, the nature of DNA substrates and cleavage products, cofactor requirements, and subunit organization of these enzymes do not appear to fit that observed with the purified nicking endonuclease in this study. Nicking endonucleases have recently captivated interest from researchers because of their applications in isothermal DNA amplification and ultrasensitive fluorescence detection method for DNA [19, 33].

## Figures and Tables

**Table 1:** Summary of the purification of the nicking endonuclease from *Streptomyces*.

Step	Volume (mL)	Protein concentration (mg/mL)	Total protein (mg)	Specific activity (U/mg)	Total units (U)	Fold purification (x)	Recovery (%)
Cell-free extract	57	11.6	661	8.62	5,700	1.0	100
0–60% NH_3_SO_4_	20	18.5	370	10.8	4,000	1.25	70
DEAE cellulose	3	5.5	16.5	54.5	900	6.0	16
Phenyl-Sepharose	1	0.2	0.2	1000	200	116	2
